# Clinical characteristics of 138 Chinese female patients with idiopathic hypogonadotropic hypogonadism

**DOI:** 10.1530/EC-17-0251

**Published:** 2017-10-10

**Authors:** Rui-yi Tang, Rong Chen, Miao Ma, Shou-qing Lin, Yi-wen Zhang, Ya-ping Wang

**Affiliations:** Department of Obstetrics and GynecologyPeking Union Medical College Hospital, Peking Union Medical College, Chinese Academy of Medical Science, Beijing, People’s Republic of China

**Keywords:** idiopathic hypogonadotropic hypogonadism, Kallmann syndrome, female, secondary sexual characteristics, fertility

## Abstract

**Objective:**

To evaluate the clinical features of Chinese women with idiopathic hypogonadotropic hypogonadism (IHH).

**Methods:**

We retrospectively reviewed the clinical characteristics, laboratory and imaging findings, therapeutic management and fertility outcomes of 138 women with IHH. All patients had been treated and followed up at an academic medical centre during 1990–2016.

**Results:**

Among the 138 patients, 82 patients (59.4%) were diagnosed with normosmic IHH and 56 patients (40.6%) were diagnosed with Kallmann syndrome (KS). The patients with IHH experienced occasional menses (4.3%), spontaneous thelarche (45.7%) or spontaneous pubarche (50.7%). Women with thelarche had a higher percentage of pubarche (*P* *<* 0.001) and higher gonadotropin concentrations (*P* *<* 0.01). Olfactory bulb/sulci abnormalities were found during the magnetic resonance imaging (MRI) of all patients with KS. Most patients with IHH had osteopenia and low bone age. Among the 16 women who received gonadotropin-releasing hormone treatment, ovulation induction or assisted reproductive technology, the clinical pregnancy rate was 81.3% and the live birth rate was 68.8%.

**Conclusions:**

The present study revealed that the phenotypic spectrum of women with IHH is broader than typical primary amenorrhoea with no secondary sexual development, including occasional menses, spontaneous thelarche or pubarche. MRI of the olfactory system can facilitate the diagnosis of KS. Pregnancy can be achieved after receiving appropriate treatment.

## Introduction

Idiopathic hypogonadotropic hypogonadism (IHH) is also known as congenital hypogonadotropic hypogonadism. This rare clinically and genetically heterogeneous disorder is characterized by complete or partial pubertal failure, which is accompanied by attenuated or normal concentrations of serum follicle-stimulating hormone (FSH) and luteinizing hormone (LH). In addition to reproductive dysfunction, patients with IHH frequently exhibit various non-reproductive abnormalities, including deafness, renal abnormalities and digital anomalies (1, 2). Furthermore, IHH can be categorized according to olfactory function as IHH with a normal sense of smell (normosmic IHH; nIHH) and IHH with anosmia/hyposmia (Kallmann syndrome; KS). Cases of KS account for approximately 40–60% of all IHH cases (3, 4).

IHH negatively affects patients’ sexual, bone and metabolic health, as well as their psychological development (5, 6). Thus, a precise early diagnosis and timely treatment are important. Previous studies have mainly focused on the molecular and genetic bases of IHH, although the genetic basis is only known for approximately 30% of the IHH cases (7). Thus, clinical examinations remain essential for the diagnosis. IHH is a disease that predominantly affects male patients, with frequencies of approximately 1:10,000 among men and 1:50,000 among women (3), which has led to women often being overlooked in previous studies. Most studies have focused on male patients, and systematic clinical research has broadened the known phenotype of male cases (8). We believe that the phenotypic spectrum of women with IHH is also broader than previously reported, although only a few large-scale studies have systematically assessed the clinical characteristics of female patients with IHH. Shaw *et al*. (9) have summarized the characteristics of 248 Caucasian female patients with IHH, and Bhagavath *et al*. (10) have reported the characteristics of 57 Caucasian female patients. The most interesting finding from previous research is that some cases may involve occasional menses, which has only been reported once (9) and has not been validated in other studies. In addition, there is no information regarding the characteristics of Asian female patients with IHH.

Peking Union Medical College Hospital is a large reproductive endocrine centre in China, and has accumulated detailed medical records from female patients who were followed up after their diagnosis of IHH. We presented the largest study to date of Asian female patients with IHH (*n* = 138) and the second largest study of female patients in the world, with detailed phenotypic profiling. We expect that our findings will help further our understanding of this disease and facilitate the early diagnosis and treatment of young female patients with IHH.

## Patients and methods

### Patients

This retrospective single-centre study evaluated all female patients with IHH who were diagnosed and treated between October 1990 and November 2016 at Peking Union Medical College Hospital (Beijing, China). Patients visiting our hospital would create medical records which were stored in medical records’ room of our hospital. We electronically searched in database using key words such as HH, IHH, nIHH or KS, and skimmed through all records of interest. At last, we identified 146 patients with a possible diagnosis of IHH. These cases were identified based on absent or incomplete pubertal development (secondary sexual characteristics and menarche) after the age of 16 years, low oestradiol (E_2_) concentrations and low or inappropriately normal serum gonadotropin concentrations (9). All patients had normal results for other pituitary hormones, prolactin, thyroid-stimulating hormone, growth hormone, cortisol and adrenocorticotropic hormone. No evidence of hypothalamic–pituitary mass lesions was observed. All patients had normal body mass index (BMI). None of the females had a known eating disorder, nor exercised excessively. However, we excluded 2 cases in which the IHH could not be distinguished from constitutional delay of growth and puberty (CDGP), 2 cases with a pituitary tumour and 4 cases with incomplete medical records. Thus, data from 138 patients were included in the current study.

Ethical approval was granted by the Ethics Committee of Peking Union Medical College Hospital. Signed informed consent forms for the treatment and data analysis were obtained from all patients or their guardians (for <18-year-old patients), and were included in the medical records.

### Clinical assessment

The patients’ medical records were carefully reviewed to retrieve data regarding their age at diagnosis (first visit and diagnosis), demographic characteristics, baseline secondary sexual characteristics, sex steroid concentrations, karyotype results, cranial magnetic resonance imaging (MRI) results, bone mineral density (BMD), bone age and pelvic ultrasonography results. Histories of anosmia or hyposmia were identified based on the patients’ self-reporting. The patients were asked to identify fragrant, pungent and offensive odours, and any cases with difficulty distinguishing between these odours were defined as anosmia. If the patients had difficulty distinguishing some of these odours, or a decreased ability to recognize odours, we defined the case as involving hyposmia. Histories regarding other diseases, familial histories, fertility outcomes and therapeutic management were also obtained from the records.

BMI was calculated as kg/m^2^. The Tanner score was used to assess baseline secondary sexual characteristics. Because our centre is a tertiary hospital, many patients were referred to our hospital after having received some hormone treatment. Information regarding secondary sexual characteristics before oestrogen treatment was obtained using the patients’ previous medical records or self-reporting. Sex hormone concentrations were all measured in our hospital’s central laboratory. The karyotype analysis was performed using cultured lymphocytes and standard Giemsa staining. Patients also underwent BMD examinations using dual-energy X-ray absorptiometry (GE Lunar Prodigy) at the lumbar spinal (L1–L4) and total hip (femoral neck). Considering the age of our patients, the results are expressed as a ‘*Z*’ score of bone density, relative to the reference data in our geographical area and matched for age, weight and race.

Each patient’s uterus was assessed using pelvic ultrasonography to obtain longitudinal and cross-sectional data. The patients were followed up for 1–9 years during the hormone treatment. In general, our patients started oestrogen at low doses and slowly increased over 1–2 years, and then provided at a maintenance dose and added a cyclic progestin. At last, we used the sequentially combined regimens with oestradiol valerate 0.5–2 mg or conjugated oestrogens 0.3–1.25 mg and adequate progesterone. The ultrasonography results were recorded annually. Data collection was performed independently by the first author (RT) and a co-author (MM). The uterine volumes and endometrial thicknesses were recorded for comparison according to the duration of oestrogen treatment. The uterine volume was calculated as 0.5233 (π/6) × uterine length (cm) × uterine width (cm) × uterine antero-posterior diameter (cm).

### Statistical methods

The data were analysed using IBM SPSS software (version 20.0 for the OS X system; IBM). Continuous variables were expressed as mean ± s.d. unless otherwise indicated, and categorical variables were expressed as number (percentage). Parametric data were compared using the independent-samples *t-*test, and non-parametric data were compared using the Wilcoxon rank sum test. The *χ* ^2^ test and Fischer’s exact test were used to test the associations of categorical variables. Differences with a *P*-value of <0.05 were considered statistically significant.

## Results

### Patient characteristics

The clinical and biochemical characteristics of the 138 patients are summarized in Table 1. The patients first presented with pubertal delay or infertility at a median age of 18 years (range: 15–37 years, interquartile range: 17–20 years). Compared to the reference ranges for healthy women, patients with IHH had significantly lower concentrations of E_2_ (12.63 ± 7.91 pg/mL, reference range (10): 25.74–61.04 pg/mL), FSH (2.25 ± 2.56 IU/L, reference range (10): 4.35–8.25 IU/L) and LH (1.33 ± 2.03 IU/L, reference range (10): 2.31–7.79 IU/L). Eighty-two patients (59.4%) were diagnosed with nIHH and 56 patients (40.6%) were diagnosed with KS. The patients with nIHH and KS generally had similar clinical characteristics, with the exception of olfactory differences and patients with KS having significantly lower BMI values (*P* = 0.045). The reduced BMI is most likely related to their reduced weight, although there was no significant difference in weight between KS and nIHH groups. The 56 patients with KS included 13 patients (23.2%) with anosmia and 43 patients (76.8%) with hyposmia. Some patients had concomitant medical conditions (Table 1). All patients had undergone renal ultrasonography, although no renal abnormalities were detected.

**Table 1:** Clinical characteristics of the 138 patients with IHH.

**Characteristic**	**All patients** (*n* = 138)	**nIHH** (*n* = 82)	**KS** (*n* = 56)	***P*value**
Age at diagnosis, years	18 (15–37)	18 (15–37)	18 (15–25)	0.409
Height, cm	162.2 ± 7.8	162.3 ± 8.5	161.9 ± 6.7	0.770
Weight, kg	55.7 ± 10.4	56.1 ± 11.3	52.6 ± 8.5	0.056
BMI, kg/m^2^	20.8 ± 3.5	21.3 ± 3.8	20.0 ± 2.8	**0.045^a^**
Concomitant medical condition, % (*N*)				
Hyperthyroidism	1.4 (2)	1.2 (1)	1.8 (1)	
Hypothyroidism	13.8 (19)	13.4 (11)	14.3 (8)	
Hyperlipidaemia	6.5 (9)	6.1 (5)	7.1 (4)	
Diabetes mellitus	0.7 (1)	1.2 (1)	–	
Hypertension	0.7 (1)	–	1.8 (1)	
Scoliosis	2.9 (4)	4.9 (4)	–	
Ventricular septal defect	1.4 (2)	2.4 (2)	–	
Uterine septum	0.7 (1)	1.2 (1)	–	
Longitudinal vaginal septum	0.7 (1)	–	1.8 (1)	
Ovarian mature teratoma^b^	0.7 (1)	1.2 (1)	–	
Ovarian endometrial cyst^c^	0.7 (1)	–	1.8 (1)	
Hearing loss^d^	0.7 (1)	1.2 (1)	–	
Depression	0.7 (1)	–	1.8 (1)	
Menarche, % (*N*)	4.3 (6)	1.2 (1)	8.9 (5)	**0.040^a^**
Thelarche, % (*N*)	45.7 (63)	48.8 (40)	41.1 (23)	0.390
Pubarche, % (*N*)	50.7 (70)	48.8 (40)	53.6 (30)	0.607
Armpit hair, % (*N*)	40.6 (56)	39.0 (32)	42.9 (24)	0.725
Sex hormone concentrations				
FSH, IU/L	2.25 ± 2.56	2.27 ± 2.63	2.24 ± 2.48	0.945
LH, IU/L	1.33 ± 2.03	1.29 ± 2.03	1.39 ± 2.03	0.778
E_2_, pg/mL	12.63 ± 7.91	11.85 ± 8.25	13.78 ± 7.30	0.161
*P*, ng/mL	0.65 ± 1.01	0.61 ± 0.66	0.71 ± 1.37	0.578
PRL, ng/mL	8.69 ± 6.20	8.64 ± 6.91	8.77 ± 5.06	0.903
*T*, ng/mL	0.29 ± 0.19	0.28 ± 0.18	0.29 ± 0.20	0.704

a A statistically significant inter-group difference (*P* *<* 0.05). ^b^The patient had undergone laparoscopic ovarian cystectomy, and a pathological examination revealed a mature ovarian cystic teratoma. ^c^The patient developed an ovarian cyst (approximately 5 cm in diameter) after hormone therapy, underwent laparoscopy and the pathological examination revealed an endometrioma. ^d^The patient has a family history of deafness, which is shown in Supplementary Fig. 1.

BMI, body mass index; E_2_, oestradiol; FSH, follicle-stimulating hormone; IHH, idiopathic hypogonadotropic hypogonadism; KS, Kallmann syndrome; LH, luteinizing hormone; nIHH, normosmic idiopathic hypogonadotropic hypogonadism; P, progesterone; PRL, prolactin; T, testosterone.

**Figure 1:**
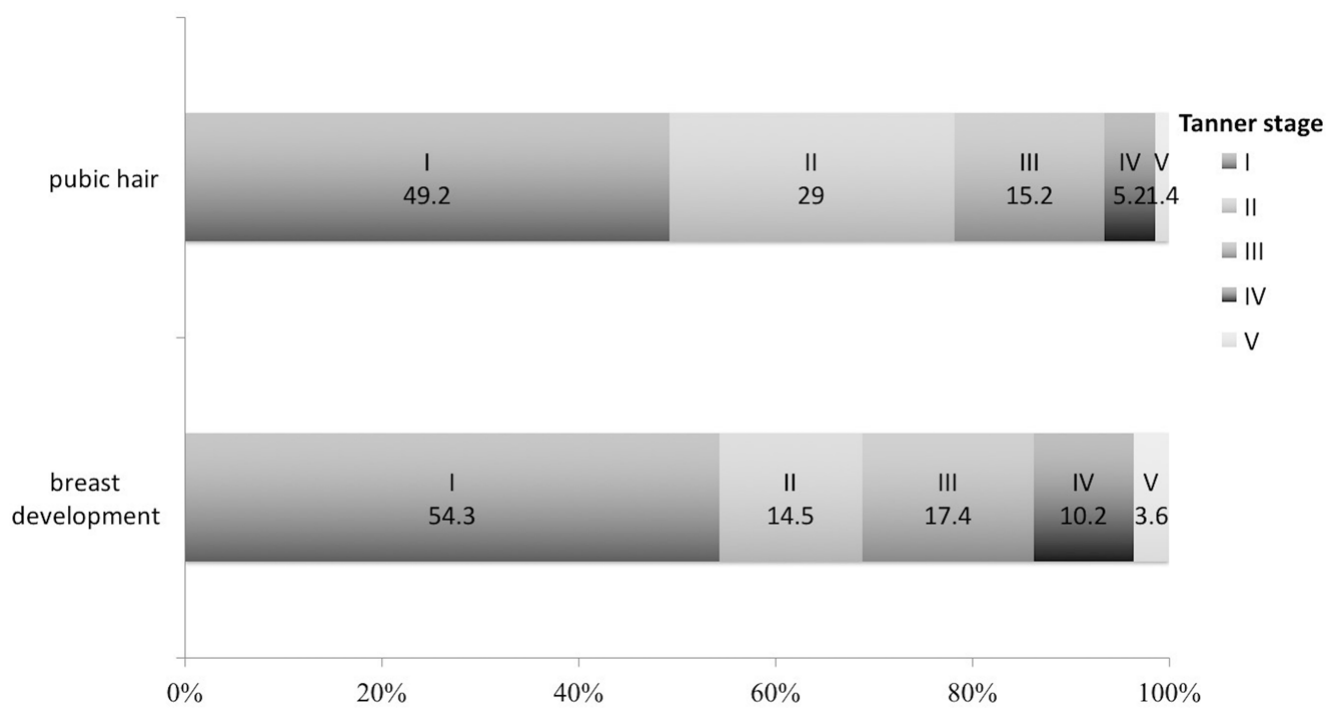
Tanner scores for breast development and pubic hair among the patients with IHH. The percentages of Tanner stages I–V are shown in different colours. IHH: idiopathic hypogonadotropic hypogonadism.

The Tanner score was used to evaluate the patients’ secondary sexual development before they received hormone therapy (Fig. 1). Spontaneous thelarche was present in 63 patients (45.7%), 70 patients (50.7%) had some degree of spontaneous pubarche and 56 women (40.6%) had sparse axillary hair. Women with thelarche were more likely to have pubarche (76.2% vs 29.3%, *P* *<* 0.001) and axillary hair growth (54.0% vs 29.3%, *P* = 0.005). Compared to patients without thelarche, patients with thelarche had higher concentrations of FSH (3.07 ± 2.99 IU/L vs 1.57 ± 1.90 IU/L, *P* = 0.001) and LH (1.92 ± 2.40 IU/L vs 0.85 ± 1.50 IU/L, *P* = 0.003), and similar E_2_ concentrations (12.60 ± 7.96 pg/mL vs 12.66 ± 7.92 pg/mL, *P* = 0.961). Compared to the 119 patients with Tanner stage I–II breast development, 19 patients with Tanner stage III–IV had higher concentrations of FSH (1.12 ± 2.13 IU/L vs 4.98 ± 3.33 IU/L; *P* = 0.001), LH (0.95 ± 1.45 IU/L vs 3.77 ± 3.18 IU/L; *P* = 0.001) and E_2_ (11.96 ± 7.25 pg/mL vs 16.87 ± 10.44 pg/mL; *P* = 0.011). Patients with or without pubarche, and with or without axillary hair, had similar biochemical characteristics. Interestingly, 4.3% (6/138) of the patients experienced one or two spontaneous menses and then developed amenorrhoea.

All 138 patients were from different families, and their family histories revealed that only one patient (0.7%) had a family member (older brother) with nIHH. If we expanded the definition to reversible delayed puberty (CDGP), isolated anosmia or isolated amenorrhoea, 29 patients (21.0%) could be considered as having a family history (Table 2). Three patients (2.2%) had twin sisters, including a sister pair that involved a KS case and a hyposmic case with normal menses, and another sister pair that involved an nIHH case and an apparently normal sister with a mother that had anosmia. In addition, one deaf patient had a family history of deafness (Supplementary Fig. 1, see section on supplementary data given at the end of this article).

**Table 2:** Family histories of the patients with IHH.

**Patient**	**Diagnosis**	**Family history**	**Affected family members**
1	nIHH	nIHH	Older brother
2	nIHH	Late menarche^a^	Mother
		Testicular dysplasia	Brother
3	nIHH	Late menarche	Grandmother
		Constitutional delay of growth	Uncle (mother’s brother), cousin
4	nIHH	Late menarche	Mother, grandmother, aunt (father’s sister)
5	nIHH	Late menarche	Mother, aunt (father’s sister)
6	nIHH	Late menarche	Mother, aunt (mother’s sister)
7	nIHH	Late menarche	Mother, sister
8	nIHH	Late menarche	Mother
9	nIHH	Late menarche	Mother
10	nIHH	Late menarche	Mother
11	nIHH	Late menarche	Mother
12	nIHH	Late menarche	Mother
13	nIHH	Late menarche	Mother
14	nIHH	Late menarche	Mother
15	nIHH	Late menarche	Mother
16	nIHH	Late menarche	Mother
17	nIHH	Late menarche	Aunt (father’s sister)
18	nIHH	Late menarche	Aunt (father’s sister)
19	nIHH	Late menarche	Aunt (father’s sister)
20	nIHH	Primary amenorrhea	Aunt (father’s sister)
21	nIHH	Premature ovarian insufficiency	Mother
22^b^	nIHH	Anosmia	Mother
23	KS	Late menarche	Mother
		Anosmia	Uncle (mother’s brother)
24	KS	Late menarche	Mother
25	KS	Late menarche	Mother
26	KS	Late menarche	Mother
27	KS	Hyposmia	Father
28	KS	Hyposmia	Father
29	KS	Hyposmia with normal menses	Twin sister

a Menarche after the age of 16 years. ^b^The patient had a twin sister who appeared completely normal.

IHH, idiopathic hypogonadotropic hypogonadism; KS, Kallmann syndrome; nIHH, normosmic idiopathic hypogonadotropic hypogonadism.

### Special examinations

#### MRI findings

High-resolution MRI images of the olfactory system were available for 32 patients with KS and 5 patients with nIHH. The MRI characteristics of the olfactory bulbs, tracts and sulci are described in Table 3. The 5 patients with nIHH had normal findings, while all patients with KS had altered rhinencephalon structures. Absence of the olfactory bulbs was noted in 28 patients with KS (28/32, 87.5%) and absence of bilateral olfactory tracts was noted in 29 patients with KS (29/32, 90.6%). The olfactory sulci were bilaterally hypoplastic in 23 patients with KS (23/32, 71.9%) and absent in 3 patients with KS (3/32, 9.4%). The 32 patients with KS included 10 anosmic cases (10/32, 31.3%) and 22 hyposmic cases (22/32, 68.7%). Nine of the 10 anosmic cases (9/10, 90.0%) and 12 of the 22 hyposmic cases (12/22, 54.5%) had bilateral agenesis of the olfactory bulb and tract, as well as hypoplastic olfactory sulci.

**Table 3:** Overview of the magnetic resonance imaging findings among patients with KS and nIHH.

	**Olfactory bulb**	**Olfactory tract**	**Olfactory sulcus**
**Diagnosis, %** (*N*)	Left/right	Left/right	Left/right
KS (*n* = 32)			
Anosmia (*n* = 10)			
9 patients (90.0)	−/−	−/−	+/+
1 patient (10.0)	−/−	−/−	−/−
Hyposmia (*n* = 22)			
12 patients (54.54)	−/−	−/−	+/+
1 patient (4.55)	−/−	−/−	−/−
1 patient (4.55)	−/−	−/−	++/++
1 patient (4.55)	++/++	−/−	−/−
1 patient (4.55)	+/+	−/−	−/+
1 patient (4.55)	+/+	+/+	+/+
1 patient (4.55)	−/+	−/++	+/+
1 patient (4.55)	−/−	++/++	−/++
2 patients (9.09)	−/−	−/−	−/+
1 patient (4.55)	−/−	−/−	+/−
nIHH (*n* = 5)			
5 patients (100)	++/++	++/++	++/++

–, absent; +, hypoplasia; ++, normal; KS, Kallmann syndrome; nIHH, normosmic idiopathic hypogonadotropic hypogonadism.

We also observed some abnormal changes among the 56 patients who underwent MRI of the hypothalamic–pituitary region. An empty sella was observed in 4 women (7.1%), including 3 patients with nIHH and 1 patient with KS. Four patients with KS and 3 patients with nIHH (12.5%) had a shrunken anterior pituitary gland.

#### BMD

The BMD values at the lumbar spine and total femur were evaluated in 61 cases (Table 4). Forty-two women received hormone treatment and 19 women were untreated. The median *Z* scores at the lumbar spine and femur were –1.20 ± 0.87 and –1.70 ± 1.06, respectively. The percentages of normal BMD (*Z* score ≥–1.0), osteopenia (–1.0 to –2.5) and osteoporosis (≤–2.5) were 28.6% (12/42), 54.8% (23/42) and 16.7% (7/42), respectively, in the hormone treatment group, compared to 5.3% (1/19), 52.6% (10/19) and 42.1% (8/19), respectively, in the untreated group. These results indicate that osteopenia was relatively common and that the BMD values increased significantly after treatment (*P* = 0.02 and *P* = 0.005, respectively).

**Table 4:** Bone mineral densities at the lumbar spine and femoral neck among patients with and without hormone treatment.

**Bone** (*Z* score)	**All patients** (*n* = 61)	With hormone treatment* (*n* = 42)	**Without hormone treatment** (*n* = 19)	***P*value**
Lumbar spine	–1.20 ± 0.87	–1.04 ± 0.87	–1.59 ± 0.75	**0.020**
Femoral neck	–1.70 ± 1.06	–1.45 ± 1.00	–2.26 ± 0.97	**0.005**

Based on the patients’ ages, the results are expressed as the *Z* score for bone density, relative to the reference data from our geographical area and matched for age, weight and race.

* The duration of treatment before DXA scan was 2.95 ± 2.14 years. Bold values indicate a statistically significant inter-group difference (*P* < 0.05).

#### Bone age

The bone ages were examined in 49 women (mean chronological age: 20.4 ± 4.5 years) before they received hormone therapy. The bone ages of 11 patients were consistent with their chronological ages (21.5 ± 6.1 years). The other 38 patients had retarded bone ages, relative to their chronological ages (20.1 ± 4.0 years), and the mean discrepancy was 4.5 ± 3.3 years.

### Uterine volumes after hormone treatment

**Figure 2:**
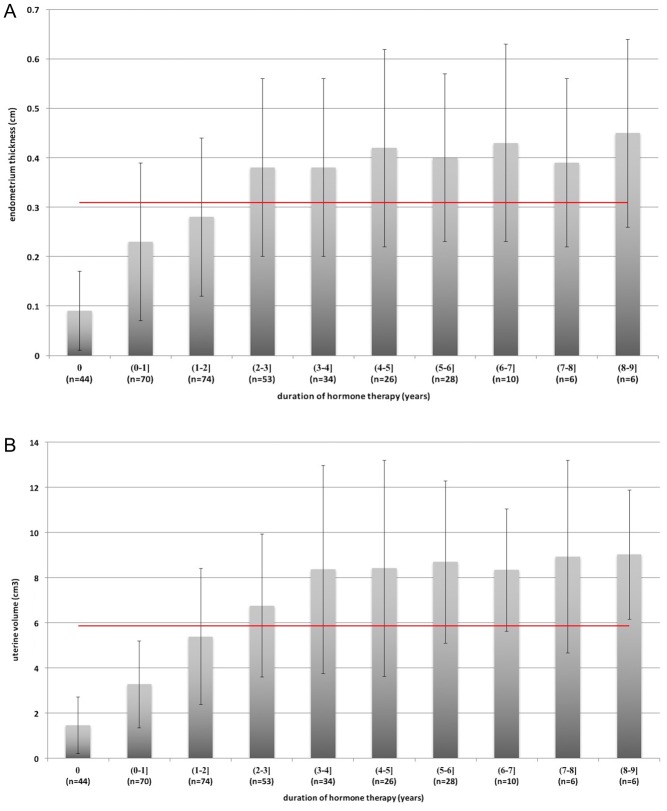
Uterine volumes and endometrium thicknesses after hormone treatment among the patients with IHH. A and B show the endometrium thicknesses and uterine volumes of patients with IHH respectively. The uterine volumes and endometrium thicknesses increased during the first 3 years of hormone treatment, and then reached a steady state. Red lines indicate the mean values and error bars indicate the standard deviations.

Several patients had received hormone replacement treatment (for several months to several years) at the time of their evaluation. The uterine volumes and endometrial thicknesses of the patients are shown in Fig. 2. During the first 3 years, prolonged hormone treatment was associated with a larger uterine volume, although the uterine volume and endometrial thickness values eventually reached a steady state. Compared to patients who achieved pregnancy with all other patients who accepted long-time hormone treatment (≥3 years), no difference was found concerning the uterine volumes (9.01 ± 3.16cm^3^ vs 8.86 ± 4.13 cm^3^, *P* = 0.871) and endometrial thicknesses (0.45 ± 0.17cm vs 0.42 ± 0.19 cm, *P* = 0.496).

### Fertility outcomes

**Figure 3:**
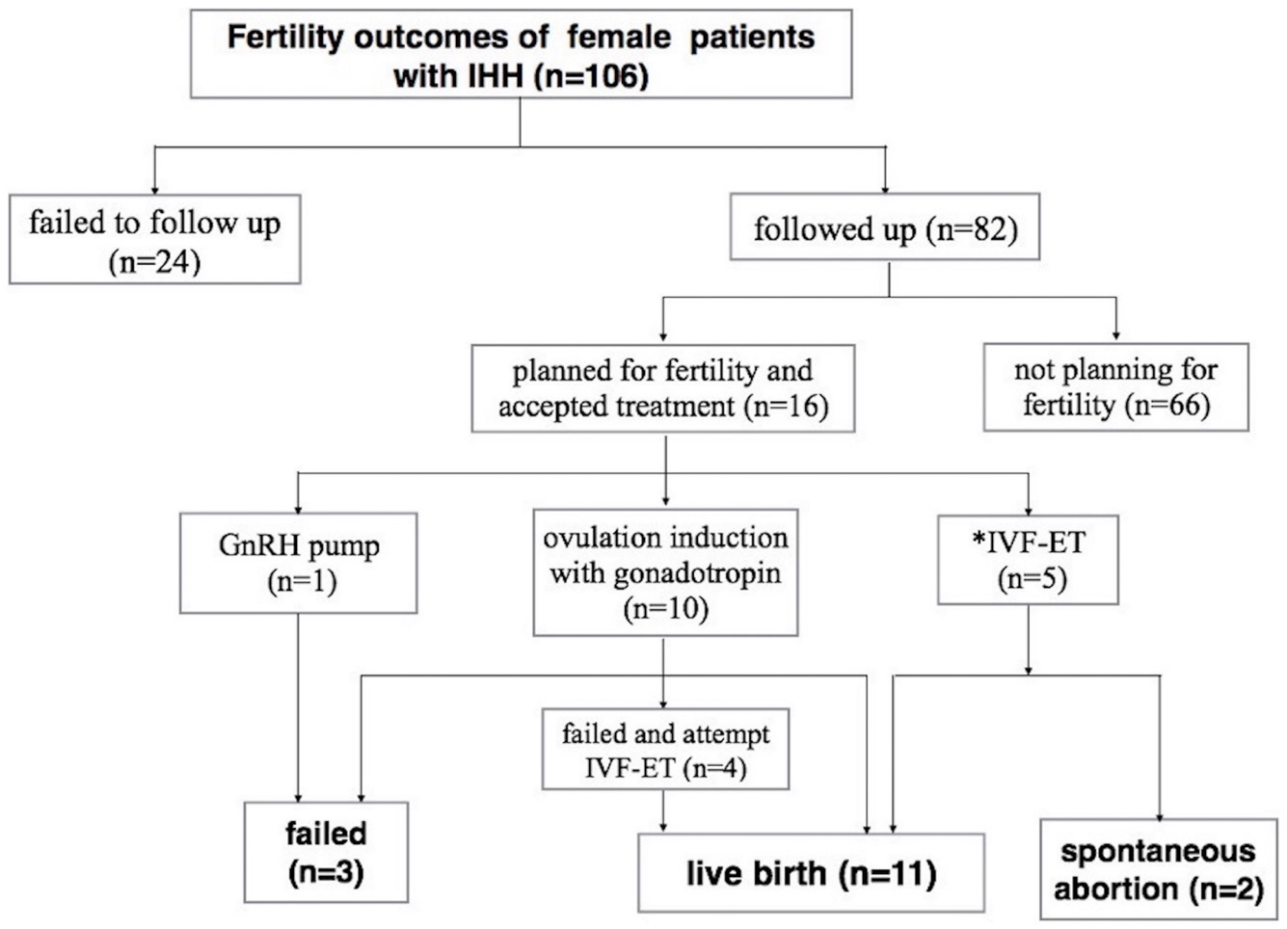
Fertility outcomes among 106 patients with IHH. Twenty-four women who were planning a pregnancy failed to attend the follow-up, according to the most recent records, and the outcomes were unclear. Eighty-two patients were followed up, including 66 patients who did not have specific plans regarding pregnancy. For the 16 patients who were seeking to conceive, 1 patient unsuccessfully underwent pulsatile gonadotropin-releasing hormone (GnRH) treatment. Ten patients underwent ovulation induction with gonadotropin, including 4 women who became pregnant and 2 women who failed and gave up further treatment. The other 4 women attempted ART and all had a successful pregnancy. Five patients directly accepted IVF because of age or male factors, including 3 women who had a live birth and 2 women who experienced first-trimester miscarriages. Thus, 81.3% of the women (13/16) became pregnant, and 68.8% (11/16) achieved a live birth. IVF-ET: *in vitro* fertilization and embryo transfer, IHH: idiopathic hypogonadotropic hypogonadism. *The five patients directly accepted IVF-ET as the first-line treatment because of age or male factors.

The fertility outcomes of 106 patients are shown in Fig. 3. Twenty-four women who were planning a pregnancy did not return for a follow-up, and their fertility outcomes were unknown according to the most recent records. Sixty-six patients did not have plans regarding fertility, which were related to both subjective and objective reasons. The subjective reasons consisted of being too young (*n* = 45), a sense of inferiority (*n* = 6) and stopped trying because of the disease (*n* = 11). The objective reasons were failed natural pregnancy and planned adoption (*n* = 4). Sixteen patients underwent fertility treatment (1 patient used a GnRH pump and 15 patients received gonadotropins or *in vitro* fertilization). The patient who received pulsatile GnRH treatment for 6 months did not become pregnant. All successful pregnancies were observed in the 15 patients who had received gonadotropins or *in vitro* fertilization (Fig. 3). The pregnancy rate was 81.3% (13/16), and 11 women achieved live births (68.8%, 11/16). These 11 women succeeded using gonadotropin treatment (*n* = 4) and/or *in vitro* fertilization and embryo transfer (IVF-ET, *n* = 7). Complications included gestational diabetes mellitus (*n* = 1) and premature delivery at 36 weeks (*n* = 1). The other 2 women experienced first-trimester miscarriages after IVF-ET.

## Discussion

The present study retrospectively analysed data from 138 women with IHH and comprehensively evaluated their clinical characteristics. As IHH negatively affects the patient’s physical and mental health, a precise early diagnosis and timely treatment are important. We mainly focused on the patients’ phenotypic features, imaging findings and therapeutic outcomes, as we expected to identify new markers to facilitate an early diagnosis. A high percentage of female patients with developed secondary sexual characteristics was found, and the possibility of occasional menses was confirmed. We also examined the findings from MRI examination of the olfactory system and provided the first report regarding the uterine volumes and endometrial thicknesses of patients with IHH.

IHH is a rare disorder that is caused by reduced GnRH production, secretion or activity, which can affect the reproductive axis. It can be subdivided into nIHH and KS, and some studies have compared these subgroups (1, 8), which are now considered two largely overlapping manifestations of a single disorder (11). The present study also revealed similar presentations for the two conditions, except the higher BMI in the nIHH group, and we summarized the features of the whole IHH group when analysing the data.

Some of our findings are in agreement with previous reports. First, we found that some patients had unique lesions that were detected during MRI, including a hypoplastic anterior pituitary gland (12, 13, 14) or empty sella (13, 15). The hypoplasia of the anterior pituitary gland may be secondary to limited stimulation from absent hypothalamic GnRH neurons (12). Second, we observed that patients with IHH experienced BMD improvement after receiving hormone treatment. The hypoestrogenic state of these patients negatively affects the peak BMD, which leads to osteoporosis and a risk of fracture (16). An early start and adequate duration of hormonal therapy may help improve BMD and reduce the risks of osteoporosis and fractures (17), although the reduced BMD may not be completely reversible. In addition, our patients presented with retarded bone ages and no karyotypic abnormalities (18).

Traditional perceptions regarding the presentations of IHH include primarily amenorrhoea and absent secondary sexual characteristics. However, recent studies have revealed that development of the breasts (19) and pubic hair (20) can be highly variable, and may be near normal in some women with IHH. In the present study, we found that approximately 50% of the cases had some degree of secondary sexual characteristic development, which is fairly high for patients with low E_2_ concentrations. It is thought that breast development depends on oestrogen concentrations, which would suggest that patients with breast development should have higher E_2_ concentrations, and that relationship is inconsistent with our results. We suspect that the spontaneous thelarche was a result of higher E_2_ concentrations, and the similar E_2_ concentrations for women with or without thelarche in the present study were most likely due to the limited sensitivity of the assay. This is because serum levels of oestradiol in female patients with IHH are often low or sometimes undetectable, and patients with breast development of Tanner stage ≥III stage had higher E_2_ concentrations, compared to patients with Tanner stage I–II.

The present study also revealed that adrenarche (pubarche and axillary hair) was not associated with gonadotrophin and E_2_ concentration. This is because adrenarche is a consequence of increased adrenal androgen production (21), and adrenarche and gonadarche are thought to be independent events (9). The present study also demonstrated that women with thelarche were more likely to undergo pubarche, which indicates that gonadal steroids may play a role in adrenarche (22). Furthermore, the aromatization of androgens may play a role in breast development.

In a large study of women with IHH, Shaw et al. were the first to observe that a small percentage of women with IHH (10%) experience occasional menses (9). We also observed that spontaneous menses occurred in 4.3% of our cases, although the cause of this finding remains unclear. We speculate that spontaneous menses reflect a temporal activation of the hypothalamic–pituitary–gonadal (HPG) axis during puberty in these patients, although there was a subsequent failure of the GnRH production. In previous reports, approximately 10–22% of the patients with IHH developed a reproductive phenomenon in which their IHH was reversed after the interruption of hormone therapy during adulthood (IHH reversal) (23). Some of the patients revert to the hypogonadal state after several years of reversal, which would indicate a relapse. The cases of reversal and relapse, as well as cases with occasional menses, are likely to have similar mechanisms that are related to frailty of the HPG axis.

Based on these findings, a subgroup of female patients with IHH can develop secondary amenorrhoea with occasional menses, as well as developed or even normal secondary sexual characteristics. These features could help identify atypical patients, but increase the complexity of the diagnosis, which remains challenging, especially among women. Unlike male neonates, there are no specific physical findings for female infants (5), and the diagnosis cannot be confirmed in most cases until puberty. CDGP is the most challenging differential diagnosis of IHH, and could have a similar presentation. It is a temporary delay of puberty because the HPG axis has not matured, and the presence of progressive pubertal development by the age of 18 years is the ‘gold standard’ for differentiation (24). Different basal and stimulation tests have been used to differentiate between adolescents, although they all have limited diagnostic specificity and sensitivity, which highlights the importance of developing additional diagnostic methods. A family history of delayed puberty is strongly suggestive of CDGP (50–75% of cases) (24), although some of our patients with IHH had a family history of CDGP, which indicates that patients with a family history of delayed puberty should undergo careful examinations and long-term follow-up to confirm the diagnosis. Another important differential diagnosis of IHH is other forms of hypogonadotrophic hypogonadism, including combined pituitary hormone deficiency, which has typical presentations of multiple pituitary deficiencies, and functional hypogonadotrophic hypogonadism (FHH) (25). Hypothalamic amenorrhoea is a common cause of FHH in females, and defined as HH with specific etiologies including excessive exercise, psychological stress or weight loss. A definite diagnosis of IHH should first exclude all these etiologies that can inhibit the gonadotrophic axis.

MRI imaging of the olfactory system is a useful diagnostic tool (12, 15), and the findings should be carefully reviewed. Underdevelopment or absence of the olfactory bulbs or sulci strongly suggests that the patient has KS, and can help differentiate between KS and nIHH (26). MRI is an especially effective diagnostic technique for pre-pubertal patients, who may not be suitable for olfactory testing (27). The present study revealed rhinencephalon alterations during the MRI scans of 32 patients with KS, and the most common findings were bilateral olfactory bulb aplasia (87.5%) and bilateral olfactory tract aplasia (90.6%). We found that MRI was 100% sensitive for detecting olfactory structure abnormalities in patients with KS, although previous reports have provided values that range from 68% to 100% (2, 12, 28, 29, 30). Interestingly, a normal olfactory system can be found in patients with a confirmed olfaction disorder (28, 31), while 7.1–37.5% of the patients with nIHH may have abnormal olfactory structures (27, 28, 29).

The present study also examined therapeutic outcomes among women with IHH. We evaluate uterine size and endometrial thickness using pelvic ultrasonography during hormone treatment, which reflects the efficacy of oestrogen treatment. Ours is the first study to collect detailed uterine volume and endometrial thickness data from a large sample of IHH cases, and we found that both uterine volume and endometrial thickness reached a steady state after approximately 3 years of hormone therapy. In addition, female patients with IHH are unable to spontaneously achieve conception, and pregnancy can only be achieved using ovulation that is induced using gonadotropins or a GnRH pump (32). The major limitations of the pulsatile GnRH pump treatment are its restriction to tertiary hospitals, high cost and negative effects on the patient’s quality of life (33). Thus, women who wish to give birth tend to prefer gonadotropin treatment with or without intrauterine insemination, or even IVF-ET. In the present study, 16 women underwent fertility treatment. Pregnancy was successfully achieved in 81.3% of these cases, and the live birth rate was 68.8%. These rates are similar to the findings of a previous study, which revealed that 7 of 9 women with IHH (77.8%) achieved a clinical pregnancy (34). Thus, female patients with IHH can have a good reproductive prognosis after receiving appropriate treatment.

## Limitations and conclusions

The present study has two important limitations. First, the retrospective design is associated with known risks of bias. Second, most patients did not undergo olfactory testing. As self-reported olfactory assessments can underestimate the true olfactory deficit (28, 29), it may be necessary to use objective smell tests (3, 35), such as the 40-item University of Pennsylvania Smell Identification Test (36). We are currently collecting additional information from our patients, especially regarding olfactory function, and we hope to perform additional analyses to address these issues.

In summary, our findings indicate that the phenotypic spectrum of women with IHH is broader than previously thought. Women with IHH can present with amenorrhoea or occasional menses, as well as absent, poorly developed or normal secondary sexual characteristics. Tanner staging should be used during the physical examination, as well as hormonal analysis, and cranial MRI can be used to diagnose IHH. Treatment for IHH aims to induce and maintain normal puberty, prevent long-term hypoestrogenism outcomes and induce fertility. Ours is the first study to obtain uterine volume and endometrial thickness data from women with IHH. We believe that the findings can help improve our understanding of the clinical characteristics of women with IHH, which may facilitate earlier diagnosis and treatment of this disease.

## Supplementary Material

Supporting Figure 1

## Declaration of interest

The authors declare that there is no conflict of interest that could be perceived as prejudicing the impartiality of the research reported.

## Funding

This work was supported by (1) CAMS Initiative for Innovative Medicine (2016-12M-1-008) and (2) the National Natural Science Foundation of China Project (grant number 81270656).
