# Supplementary figures and images for: Clinical characteristics of 138 Chinese female patients with idiopathic hypogonadotropic hypogonadism
Source: Endocr Connect. 2017 Oct 10;6(8):800–10. doi: 10.1530/EC-17-0251 (PMC5682410; doi:10.1530/EC-17-0251)

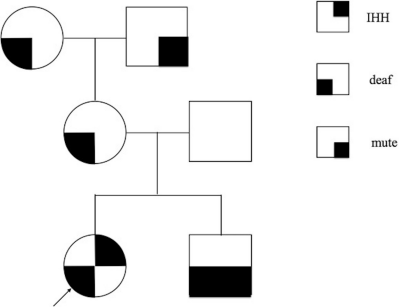

Supplement: Supporting Figure 1 [file ec-6-800-s001.pdf]
